# Traditional Chinese medicine in the treatment of diabetic kidney disease: the mechanisms of signaling pathways regulations

**DOI:** 10.3389/fendo.2026.1792000

**Published:** 2026-02-25

**Authors:** Wencan Li, Chaoqi Lei, Qinxuan Wu, Keqian Chen, Zheng Liu, Qichang Xing, Xiang Liu, Jiani Zhang

**Affiliations:** 1 Department of Clinical Pharmacy, The Central Hospital of Xiangtan (The Affiliated Hospital of Hunan University), Xiangtan, Hunan, China; 2 Department of Traditional Chinese Medicine, The Central Hospital of Xiangtan (The Affiliated Hospital of Hunan University), Xiangtan, Hunan, China; 3 Hunan Provincial Key Laboratory of the Research and Development of Novel Pharmaceutical Preparations, the “Double-First Class” Application Characteristic Discipline of Hunan Province (Pharmaceutical Science), Changsha Medical University, Changsha, Hunan, China

**Keywords:** diabetic kidney disease, diabetic nephropathy, mechanism, signaling pathway, traditional Chinese medicine

## Abstract

Diabetic kidney disease (DKD) represents a diabetes-driven microvascular complication characterized by renal physiological and metabolic disorders and is considered a top-ranking trigger of progression to end-stage renal disease and death in diabetic patients. Although the drugs commonly used in clinical practice for DKD provide some renal protection, their toxic side effects and limited ability to halt further progression of the disease remain unsatisfactory. Traditional Chinese medicine (TCM) brings centuries of accumulated practice and distinct therapeutic strengths to the management of chronic diseases with complex pathogenesis such as DKD. Its characteristics of multi-target and multi-pathway intervention establish a solid material basis for DKD therapy, while its low toxicity profile aligns well with the chronic nature of DKD. This review elaborates on the therapeutic potential of examining TCM’s impact on DKD through the prism of cellular signaling pathways and reveals that the Nrf2, TGF-β/Smad, NF-κB/NLRP3, MAPK, and PI3K/AKT signaling pathways warrant particular attention in the TCM treatment of DKD. The TCM agents involved include astragaloside IV, Shengqing Jiangzhuo formula, Huangkui capsule, Fuxin granules, Liuwei Dihuang pill, *Taxus chinensis*, Burdock fructooligosaccharide, baicalin, hirudin, rutin, and fermented seaweed extracts. This review aims to refresh and consolidate the signaling pathways engaged by TCM in DKD, sift for promising targets and drugs, and supply both conceptual and experimental scaffolding for future anti-DKD therapeutics.

## 1 Introduction

**Figure 1:**
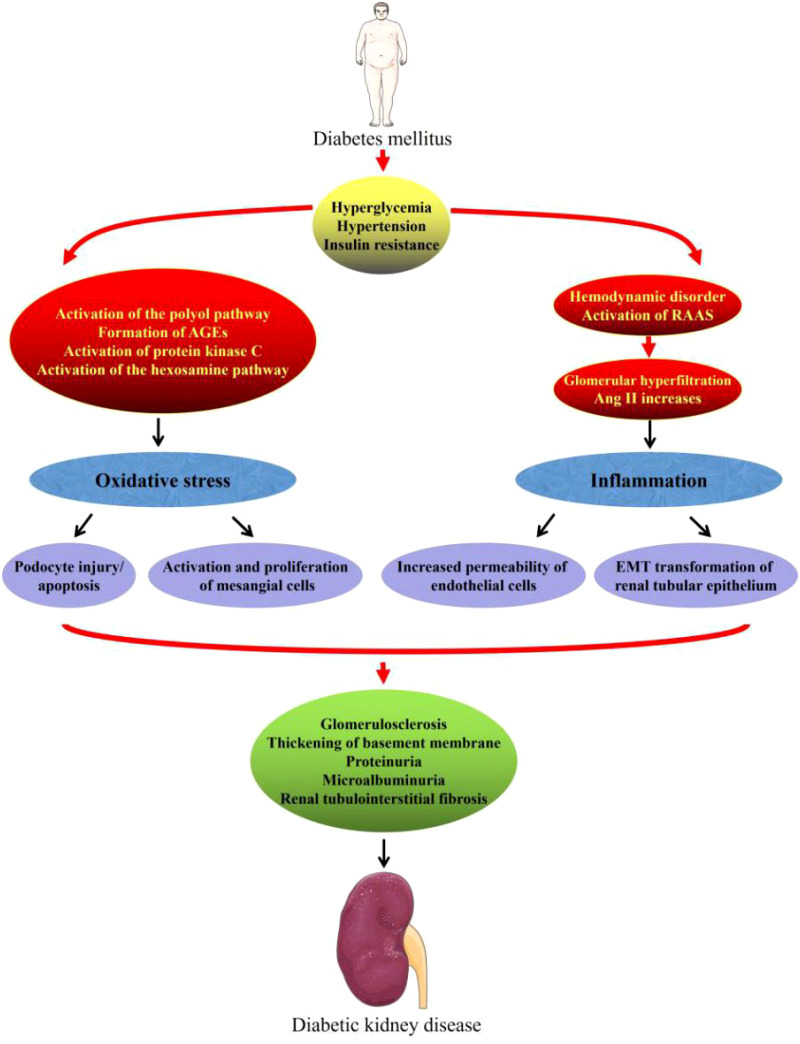
Mechanism of the occurrence and development of DKD.

**Figure 2:**
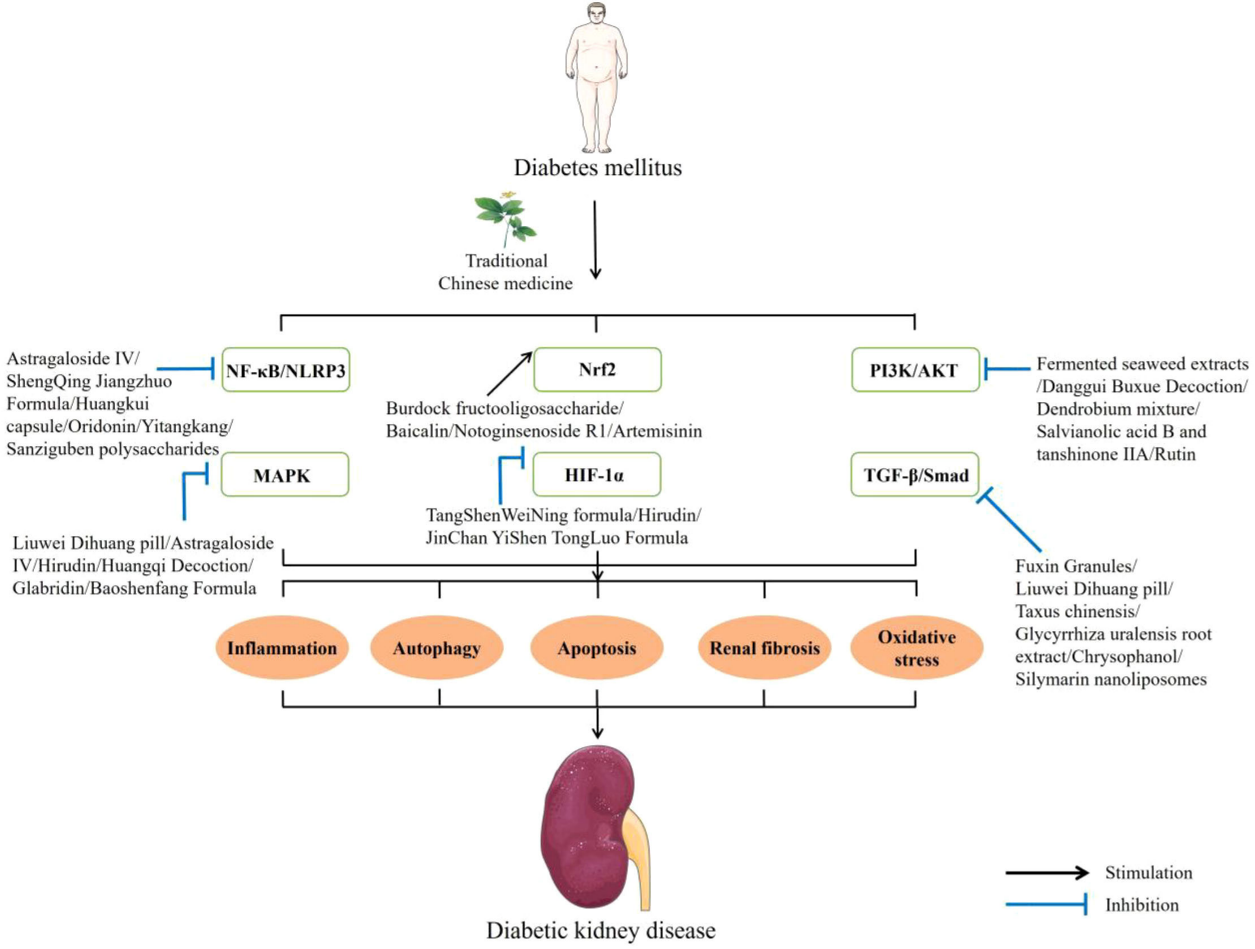
Candidate DKD-related signaling pathways modulated by TCM.

The number of people with diabetes continues to surge and is projected to exceed 780 million by 2045. Persistent hyperglycemia in diabetes leads to endocrine dysregulation and metabolic disorders, eventually progressing to diabetic complications that impose formidable challenges on patients’ health and family finances—an issue that has become a focus of global debate (1–3). Diabetic kidney disease (DKD) represents a diabetes-driven microvascular complication characterized by renal physiological and metabolic disorders and is considered a top-ranking trigger of progression to end-stage renal disease and death in diabetic patients. The pathogenesis of DKD is highly complex. Persistent hyperglycemia acts as a core initiator that gradually induces metabolic disorders of glucose and lipids through a cascade of interactions with insulin resistance. Subsequently, it synergistically activates multiple classic metabolic pathways such as the polyol pathway, advanced glycation end products (AGEs) formation, protein kinase C (PKC), and hexosamine pathway. At the same time, the abnormal renal hemodynamics caused by hyperglycemia can activate the renin–angiotensin–aldosterone system (RAAS), and the interaction between them forms a vicious cycle. The cross-regulation of these various abnormal processes directly leads to an increase in reactive oxygen species (ROS) production and an elevation in oxidative stress levels, ultimately causing oxidative stress damage to the kidneys. In addition, damage to renal cells caused by hyperglycemia can trigger the release of inflammatory mediators, leading to the recruitment and infiltration of macrophages, monocytes, and activated T lymphocytes into renal tissue. This series of pathological physiological cascades can mediate the damage of intrinsic renal cells and trigger cell apoptosis and destruction of renal tissue morphology and structure, ultimately resulting in glomerular sclerosis and tubulointerstitial fibrosis and further promoting the progression of DKD (4–6) (**Figure 1**). The principal pathological hallmarks of DKD are expansion of the glomerular basement membrane together with deposition of proteins and other extracellular matrix, fibrosis of the renal tubule, expansion of the mesangial matrix, and dysfunction or depletion of the podocytes. Clinically, it manifests as persistent proteinuria, a progressive decline in glomerular filtration rate, rising serum creatinine, and impaired renal function that ultimately progresses to kidney failure (7–9). At present, the pharmacological treatment of DKD in the clinic relies mainly on renin–angiotensin–aldosterone system inhibitors; however, their toxic side effects and limited ability to halt the further progression of DKD remain unsatisfactory (3, 10). Traditional Chinese medicine (TCM) has held a pivotal position in clinical diagnosis and treatment in China for centuries and serves as the primary avenue through which individuals safeguard against and address ailments. Emerging pre-clinical evidence indicates that TCM exerts marked renoprotective effects against DKD.​Unlike single-target synthetic agents, it is characterized by multiple components that converge on several targets simultaneously, amplifying the therapeutic impact through coordinated synergy. Especially in the process of treating chronic diseases, its characteristic of having few adverse reactions also reduces the harmful effects on the patients themselves (10, 11). In recent years, as researchers worldwide have become increasingly interested in TCM, a growing body of studies on TCM against DKD has emerged. Regrettably, a comprehensive and critical review of TCM for DKD is still lacking. We hope that this review will provide new ideas and experimental evidence to support the creation of future anti-DKD drugs (**Figure 2**).

## 2 TCM modulates DKD in the signaling pathway

### 2.1 NF-κB/NLRP3 signaling pathway

Nuclear factor kappa B (NF-κB) stands as a master transcriptional switch in cellular signaling, which is involved in multiple processes such as cell transformation, survival, proliferation, invasion, angiogenesis, metastasis, and inflammation. In DKD, the NF-κB signaling pathway is activated in the kidneys and can be activated by various stimuli including high glucose, advanced glycation end products (AGE), inflammatory factors, chemokines, and oxidative stress, which is crucial in mediating cellular stress, driving inflammatory responses, and promoting apoptosis within renal tissues. The NF-κB family comprises five members: RelA/p65, RelB, c-Rel, NF-κB1/p50, and NF-κB2/p52. They are encoded by the corresponding genes RELA, RELB, REL, NFKB1, and NFKB2. In the resting state, these proteins exist as complexes bound to the inhibitor of NF-κB (IκB). Upon extracellular stimulation (for example, IL-6, iNOS, and ICAM-1 of TNF-α), the signal is transduced via adaptor proteins to the IκB kinase (IKK) complex, which phosphorylates and ubiquitinates IκB, thereby promoting its degradation. Consequently, NF-κB is liberated and translocates into the nucleus to initiate transcription. Nucleotide-binding domain and leucine-rich repeat receptor containing a pyrin domain 3 (NLRP3) inflammasome are a complex composed of NLRP3, apoptotic speck-like protein (ASC), and pro-caspase-1. Diabetes causes disorders in lipid and glucose metabolism, which leads to elevated levels of oxidized low-density lipoprotein and glycosylated low-density lipoprotein. This could induce macrophages to bubble or infiltrate the vascular wall, transform into a pro-inflammatory phenotype, and activate NF-κB. After NF-κB enters the nucleus, it can act as an initiating signal to induce the activation of the NLRP3 inflammasome, thereby cleaving pro-caspase-1 into caspase-1 and promoting the release of downstream inflammatory factors interleukin (IL)-1β and IL-18. The release of these inflammatory factors further leads to the over-expression of various cytokines and chemokines, causing the inflammation of renal tissues and promoting the infiltration of white blood cells and macrophages, resulting in renal injury and increased microalbuminuria in DKD (12–15).

*Astragalus membranaceus* is one of the most commonly used TCM, which has biological activities such as activating immune mechanisms, reducing oxidative stress, alleviating inflammation, and lowering blood glucose (16, 17). Astragaloside IV (AS-IV) is the main constituent of *A. membranaceus* and has been shown to be applicable to multiple illnesses comprising DKD. He et al. (18) reported the mechanism of action of AS-IV against DKD. AS-IV reduces the levels of triglycerides (TG) and total cholesterol (TC) in the diabetic rat model induced by a high-fat diet coupled with streptozotocin (STZ), and renal lipopathy showed dose-dependent remission. AS-IV could also reduce the levels of urine albumin–creatinine ratio, serum creatinine (SCr), and blood urea nitrogen (BUN). AS-IV was able to reverse significant renal basement membrane expansion and extensive podocyte fusion in diabetic rats in oil-red O staining experiments. These data indicate the ability of AS-IV to improve renal function impairment in diabetic rats. Klotho, an anti-aging protein secreted in the distal convoluted tubules of the kidneys, has many functions encompassing anti-aging effects. AS-IV upregulates the expression of klotho and nephrin in the serum and kidneys of diabetic rats. In podocytes induced by high glucose (HG), AS-IV could upregulate the expressions of nephrin and klotho in damaged podocytes, and it improves podocyte bone fractures, morphological changes, and cell collapse. Furthermore, AS-IV could reduce the expression levels of caspase-1, IL-1β, IL-18, GSDMD-N, NLRP3, apoptosis-associated speck-like protein (ASC), p-p65, and p65 *in vivo* and *in vitro*. Notably, when the klotho protein was eliminated, the inhibitory effect of AS-IV on NLRP3 was significantly diminished, suggesting that overexpression of klotho may inhibit and block the expression of the NLRP3 inflammasome complex. The above-mentioned results indicate that the protective effect of AS-IV in upregulating klotho expression in diabetes-induced podocyte injury is related to the NF-κB/NLRP3 signaling pathway.

ShengQing Jiangzhuo formula (SJF) is a TCM formula that consists of *Astragalus membranaceus*, *Smilax glabra* Roxb., *Rheum officinale*, *Bombyx batryticatus*, *Periostracum cicadae*, and *Rhizoma curcumae* Longae, which functions in protecting the vital energy, regulating the qi movement, transforming turbid toxins, and unblocking the kidney meridians (19). Tan et al. (19) found that HE staining of renal tissues in db/db mice showed significant glomerular hypertrophy, diffuse thickening of the capillary basement membrane, disorder of renal tubular epithelial cells accompanied by vacuolar degeneration, inflammatory cell infiltration in the renal interstitial tissue, and an increased positive rate of TUNLE staining, suggesting nuclear damage. After SJF intervention, all of these pathological changes improved to varying degrees. In addition, the levels of urine protein (UTP), SCr, IL-1β, and IL-18 in the blood of db/db mice all increased to varying degrees, while the intervention of SJF could reverse their increases. Furthermore, in a further mechanism research, the expression levels of IL-1β, IL-18, transforming growth factor-β1 (TGF-β1), TLR4, p-p65, MyD88, NLRP3, caspase-1, and GSDMD-N in the renal tissues of db/db mice were significantly increased, and SJF could downregulate their expression levels. The above-mentioned results indicate that SJF could improve DKD by suppressing the TLR4/NF-κB/NLRP3 signaling pathway, reducing the inflammatory cascade of renal tissue and alleviating renal injury.

Huangkui capsule (HKC), extracted from *Abelmoschus manihot* (L.) Medic, is often used in clinical practice for the treatment of chronic kidney disease (20). Han et al. (20) found that HKC could improve general conditions and biochemical parameters such as kidney weight (KW), urinary albumin (UAlb), serum creatinine (SCr), and serum albumin (Alb) in DKD rats. Immunohistochemical and Western blot analysis results showed that HKC could reduce the expression levels of vimentin, collagen-1, and α-smooth muscle actin (α-SMC), demonstrating its ability to attenuate renal tubular epithelial-to-mesenchymal transition (EMT) in DKD rats. Furthermore, in further mechanistic studies, it was found that the expression levels of NLRP3, caspase-1, IL-1β, TLR4, p-IKK, and p65 were upregulated in the renal tissue of DKD rats, which were significantly downregulated after HKC intervention. These results suggest that HKC has an effect on alleviating renal EMT in DKD, and this effect is associated with the TLR4/NF-κB/NLRP3 signaling pathway.

Additionally, oridonin (21), Sanziguben polysaccharides (22), and Yitangkang (23) have shown significant effects in alleviating DKD by a mechanism related to the inhibition of the NF-κB/NLRP3 signaling pathway.

### 2.2 TGF-β/Smad signaling pathway

TGF-β is a highly pleiotropic cytokine that is widely expressed in multiple human organs, including the kidney. In the course of DKD, chronic hyperglycemia first induces TGF-β activation, which stimulates renal cells to secrete extracellular matrix (ECM) proteins that are deposited as connective tissue. Once this tissue becomes excessive, the resulting fibrosis causes organ scarring that disrupts renal structure and function (1, 24). TGF-β1, TGF-β2, and TGF-β3 are the principal isoforms of TGF-β, and they signal through three corresponding receptors: TGF-βRI, TGF-βRII, and TGF-βRIII. TGF-β initiates intracellular signaling by binding to TGF-βRII, thereby activating the kinase activity of TGF-βRI. Smad proteins serve as the core messengers of the TGF-β/Smad signaling pathway. Once Smad2/3 are phosphorylated by the activated TGF-βRI kinase, they associate with Smad4 to form a heteromeric complex that translocates into the nucleus to regulate target-gene expression (1, 24, 25).

Fuxin granules (FXG) are a clinical empirical formula for the treatment of DKD which is composed of seven TCM and has the effects of improving kidney disease and protecting blood vessels (26). Zheng et al. (26) found that FXG could reduce the elevated blood glucose and lipid levels in db/db mice. Thickened GBMs, severe glomerulosclerosis and tubular vacuolar degeneration, tubulointerstitial fibrosis, and lipid droplet deposition were clearly found in HE, PAS, and Masson staining experiments, and the above-mentioned indicators improved to varying degrees after FXG intervention, suggesting that FXG ameliorated the renal micro-architecture and robustly halted renal fibrosis progression. In addition, FXG reduced the BU, SCr, UCr, and mALB levels in the detection of kidney-function-related parameters of db/db mice. These results indicate that FXG improved the glomerular filtration function. Furthermore, further mechanism studies have shown that FXG reduced the expression levels of TGF-β1, p-Smad2/3, Smad2/3, VEGFA, and VEGFR2 and increased the expression level of eNOS. The above-mentioned results indicate that FXG improved hyperglycemia and hyperlipidemia in db/db mice, optimized the structure and function of the kidneys, and thereby alleviated the symptoms of DKD. These effects are potentially associated with the regulation of TGF-β1/Smad and VEGF/VEGFR2 signaling pathways.

Liuwei Dihuang pill (LWDHP) from prepared *Rehmannia* root, *Cornus officinalis*, Cortex Moutan, Rhizoma Dioscoreae, Poria Cocos, and Alismatis Rhizoma is composed of six TCM ingredients. They have the therapeutic effects of nourishing Yin and tonifying the kidney, benefiting essence and marrow in TCM, and have beneficial effects in chronic inflammation, oxidative stress, and DKD (27, 28). Zhong et al. (28) found that the total protein (TP), BUN, and Cr of STZ-induced DKD rats were significantly increased, while these indicators were decreased and renal function was improved after LWDHP administration. The results of HE staining and Masson collagen staining revealed that LWDHP could improve renal fibrosis and reduce the calculated signal value per area of renal tissue in DKD rats. Furthermore, electron microscopy examination revealed thickening of capillary basement membranes and matrix deposition in the mesangial area in DKD rats, and LWDHP could ameliorate these pathological alterations. Moreover, LWDHP reduced the level of malondialdehyde (MDA) and increased the levels of SOD and NOS in the renal tissue of DKD rats, suggesting the antioxidant stress effect of LWDHP. In deeper mechanistic work, LWDHP could also reduce the expression levels of TGFβ-RI, TGFβ-RII, p-Smad2/3, α-SMA, NF-κB, p-p38, and p-ERK in the renal tissues of DKD rats. The above-mentioned findings suggest that LWDHP has the potential to improve renal fibrosis and alleviate the development of DKD through anti-oxidative stress and reduction of lipid peroxidation damage. These effects are related to the inhibition of TGF-β/Smad, NF-κB, and MAPK signaling pathways.

*Taxus chinensis* (TCN) is a precious TCM whose main medicinal parts are branches with leaves. The active ingredients it contains have diverse bioactivities, including antioxidation, anti-tumor, and anti-diabetic complications (29–31). Weng et al. (31) documented the therapeutic impact of TCN on high-fat diet coupled with STZ-induced diabetic rats. Studies have found that the body weight of diabetic rats has decreased, and the blood glucose level has increased. However, these changes could be reversed after TCN intervention. In terms of renal function, TCN could reduce the levels of BUN, SCr, and urinary albumin excretion rate (UAER) in diabetic rats and ameliorate the renal function of diabetic rats. Furthermore, the kidneys of diabetic rats indicated glomerular atrophy, mesangial dilatation, tubular dilatation, and inflammatory cell infiltration. The intervention of TCN could significantly improve these renal histopathological damages. Mechanistically, TCN significantly reduced the expression levels of TGF-β1, p-Smad2/3, and α-SMA in the renal tissues of diabetic rats. The above-mentioned findings indicate that TCN could alleviate DKD by reducing renal injury and improving renal function. The mechanism may be associated with the inhibition of the TGF-β1/Smad signaling pathway.

In addition, *Glycyrrhiza uralensis* root extract (32), chrysophanol (33), and silymarin nanoliposomes (34) have demonstrated marked DKD alleviation via mechanisms linked to the inhibition of the TGF-β/Smad signaling pathway.

### 2.3 Nrf2 signaling pathway

Nuclear factor erythroid 2-related factor 2 (Nrf2) is a pivotal transcription factor that safeguards cellular redox homeostasis, defends against stress, and dampens inflammatory signaling. Studies have demonstrated that Nrf2 holds great therapeutic potential for renal diseases in both humans and animals (35, 36). Under redox-balanced conditions, Nrf2 is predominantly sequestered in the cytoplasm as a complex with Kelch-like ECH-associated protein 1 (KEAP1). When this balance is disrupted and oxidative stress ensues, accumulation of ROS triggers dissociation of the complex, and Nrf2 is then released, moves into the nucleus, and triggers the transcription of target genes involved in antioxidant defense, xenobiotic metabolism, cellular bioenergetics, and inflammatory regulation (35–37).

As mentioned earlier, SJF could attenuate renal inflammation through the suppression of the TLR4/NF-κB/NLRP3 signaling pathway, thereby playing a role in improving DKD. Yu et al. (38) also reported the intervention effect of Shengqing Jiangzhuo capsule (SJC) on DKD. SJC could reduce the body weight and blood glucose levels of db/db mice and decrease the levels of SCr, urine microalbumin, AGEs, ROS, TC, TG, and LDL-C while increasing the levels of HDL-C in the serum of db/db mice. The experimental results of HE, Masson, and PAS staining could be observed, and db/db mice presented with glomerular enlargement, mesangial expansion, tubular dilation, vacuolation loss of tubular epithelial cells, and glycogen buildup, and these pathological indicators were improved after intervention with SJC. These results suggest the effectiveness of SJC in terms of renal function and structure in db/db mice. In the mechanism study, SJC could upregulate the expression levels of Nrf2, γ-GCS, SOD1, HO-1, NQO1, and GST and downregulate the expression level of KEAP1 in the renal tissues of db/db mice. Similarly, in further *in vitro* studies, the intervention results of SJC on the above-mentioned indicators also showed consistency. All of the above-mentioned results indicate that SJC could provide protection to DKD by activating the Keap1/Nrf2 signaling pathway.

Burdock fructooligosaccharide (BFO) is a high-purity fructose extracted from burdock root, with immunomodulatory, anti-inflammatory, and anti-diabetic biological activities (39–41). Zhu et al. (41) found that BFO decreased the levels of UTP, SCr, BUN, TC, TG, and LDL-C and increased the levels of HDL-C, SOD, and catalase (CAT) in high-fat diet coupled with STZ-induced DKD mice. These findings underscore the antioxidant capacity of BFO and its protective effect on renal function. In the histopathological analysis of renal tissue, HE, PAS, and Masson staining methods were used. It was found that in DKD mice, mesangial cell proliferation, cytoplasmic vacuolation, glomerular hypertrophy, significant thickening and widening of the basement membrane of renal tubules, and abundant inflammatory infiltrates were observed, and after intervention of BFO, the above-mentioned histopathologic abnormalities improved. Furthermore, BFO upregulates the expression levels of Nrf2, HO-1, and Bcl-2 and downregulates the expression levels of Bax in DKD mice. The above-mentioned results indicate that BFO could improve renal function by regulating the Nrf2/HO-1 signaling pathway, thus exerting a therapeutic effect in DKD.

Baicalin is a flavonoid compound isolated and extracted from the well-known TCM plant *Screenaria baicalensis* Georgi and is also its main metabolite. It has biological activities such as anti-inflammation, anti-oxidation, anti-cancer, and anti-diabetes (42–44). Ma et al. (44) found that baicalin reduced the urine albumin level, urine albumin/creatinine ratio (ACR), and urine albumin excretion rate (AER) and alleviated glomerular hypertrophy and mesangial matrix expansion, improving podocyte detachment, foot process effacement, and GBM thickening in db/db mice. These effects of baicalin suggest its ability to improve albuminuria and tissue structural alterations in DKD mice. The expression levels of pro-apoptotic proteins Bax and cleaved caspase-3 increased, while the expression level of anti-apoptotic protein Bcl-2 decreased in DKD mice. Their expression could be reversed after intervention by using baicalin. It is suggested that baicalin could inhibit cell apoptosis in DKD. In addition, baicalin could increase the levels of glutathione peroxidase (GSH-PX), SOD, and CAT and reduce the level of MDA in DKD mice, indicating that baicalin could alleviate oxidative stress damage by enhancing the activity of antioxidant enzymes. Mechanistically, baicalin significantly upregulated the protein expression levels of Nrf2, HO-1, and NQO-1. These findings suggest that baicalin could alleviate oxidative stress by activating the Nrf2 signaling pathway, thereby playing a role in improving DKD.

In addition, notoginsenoside R1 (45) and artemisinin (46) have markedly ameliorated DKD through mechanisms anchored in suppressing the Nrf2 signaling pathway.

### 2.4 MAPK signaling pathway

Mitogen-activated protein kinase (MAPK) consists of a group of serine/threonine protein kinases, and it can be divided into three subfamilies: extracellular regulated kinase 1/2 (ERK1/2), p38 MAPK, and c-Jun N-terminal kinase (JNK), and they are activated by inflammatory cytokines, bacterial products, HG conditions, and other stimuli, thereby controlling many aspects of cellular function. When MAPK is triggered by external signals, MAPK3 is first phosphorylated, which subsequently activates MAPK2. MAPK2 then phosphorylates MAPK itself. Activated MAPK is translated into the nucleus and phosphorylates transcription factors to regulate diverse responses. The MAPK signaling pathway serves as a common intersection for signal transduction involving inflammation, oxidative stress, and apoptosis, and its dysregulation is closely associated with the onset and progression of chronic kidney diseases, including DKD (47–50).

AS mentioned earlier, AS-IV could upregulate the expression of klotho by regulating the NF-κB/NLRP3 signaling pathway, thereby playing a role in improving DKD. Song et al. (51) found that AS-IV reduced the levels of UAlb, UACR, and SCr and increased the level of HDL-C in STZ-induced diabetic mice. Furthermore, diabetic mice presented larger glomerular tuft areas (GTA), higher mesangial matrix, and greater podocyte foot process width (FPW). The intervention of AS-IV could improve the above-mentioned pathological changes. In further mechanism studies, AS-IV was able to reduce the expression levels of p-MEK1/2, p-ERK1/2, and p-RSK2. The above-mentioned experimental results indicate that AI-IV could improve the abnormalities of renal structure and function by inhibiting the MEK1/2-ERK1/2-RSK2 signaling pathway, thereby playing a role in improving DKD. Similarly, as mentioned earlier, baicalin could improve DKD through oxidative stress mediated by the Nrf2 signaling pathway. In addition, Ma et al. (44) found, in an in-depth study of the mechanism of action of baicalin, that it reduced the expression levels of IL-1β, IL-6, MCP-1, TNFα, p-Erk1/2, p-JNK, and p-p38. It is suggested that baicalin could improve DKD by inhibiting inflammation mediated by the MAPK signaling pathway.

Leeches were first documented in *Shen Nong Ben Cao Jing* and are said to break up blood, dissipate blood stasis, and dredge the collaterals. Hirudin is a principal bioactive isolate derived from the salivary glands of leeches, which has anticoagulant, antifibrotic, antithrombotic, and anti-inflammatory effects and also has potential benefits for the treatment of DKD (52, 53). Han et al. (54) found that the kidney weight/body weight, SCr, BUN, and proteins in the urine increased significantly in STZ-induced DKD rats. However, the levels of these biochemical indicators in the kidneys could be reduced after hirudin intervention. In addition, in HE staining, TUNEL staining, and quantitative pathological analysis, glomerular bulging, mesangial cell proliferation, mesangial thickening, small renal capsule sac cavity, increased apoptotic cells, and an elevated glomerular area could be observed in DKD rats—all of these indicators were improved to varying degrees after hirudin intervention. In further studies, hirudin reduced the expression levels of p-p38, p-p65, TNF-α, IL-1β, and IL-6 in the renal tissues of DKD rats, and these results were further confirmed in HG-induced podocytes. The above-mentioned results indicate that hirudin could protect DKD by restraining the p38 MAPK/NF-κB signaling pathway to attenuate inflammatory responses and podocyte apoptosis, thereby improving renal injury.

In addition, Huangqi decoction (55), glabridin (56), and Baoshenfang formula (57) have markedly ameliorated DKD through mechanisms anchored in suppressing the MAPK signaling pathway.

### 2.5 PI3K/AKT signaling pathway

The phosphoinositol-3 kinase (PI3K)/AKT signaling pathway is a key signaling pathway in kidney diseases and functions as a critical determinant across a broad range of cellular activities. PI3K is an intracellular phosphatidylinositol kinase composed of regulatory subunit p55/p85 and catalytic subunit p110, possessing both protein and lipid kinase activities. PI3K could be classified into classes I, II, and III based on its molecular architecture, activation pattern, and choice of substrates. Among them, class I PI3K (p85/p110) is the most frequently studied. After upstream signaling, the p110 catalytic subunit could catalyze related substrates to recruit AKT and activate it. Subsequently, AKT exerts its biological functions by regulating cell survival, inflammation, oxidative stress, cell proliferation, and apoptosis (11, 48, 58, 59).

Rutin is a polyphenolic flavonoid that is widely present in many TCM and foods and has powerful antioxidant and anti-inflammatory properties (60, 61). Dong et al. (62) found that rutin reduced the levels of urinary creatinine (UCr), SCr, BUN, and uric acid (UA) in db/db mice, pointing to rutin’s renoprotective benefit. In HE, PAS, and Masson staining analysis, dilated renal tubules, hypertrophy of the glomerulus, and deposition of collagen occurred in the kidneys of db/db mice, and rutin could improve the above-mentioned kidney injuries. Furthermore, rutin could upregulate the expression of CD31, vascular endothelial cadherin (VE-cadherin), syndecan-1, and glypican-1 and downregulate the expression of α-SMA and collagen 1. The results suggest that rutin could attenuate endothelial-to-mesenchymal transition (EndMT) and restore the damaged charge barrier. In further mechanism studies, rutin upregulated the expression levels of LC3BII/LC3BI ratio and Beclin1 and downregulated the expression levels of HDAC1, p-PI3K, p-AKT, p-mTOR, and p-S6K1, and it could also increase the number of autophagosomes. The above-mentioned data indicate that rutin could attenuate EndMT by inhibiting HDAC1 in DKD and restoring autophagy, and this mechanism of action may be associated with the PI3K/AKT/mTOR signaling pathway.

Seaweed is a precious marine resource, and one of the TCM that are both edible and medicinal. It possesses biological activities such as in reducing blood glucose, reducing blood lipid, anti-inflammation, and antioxidation (63–65). Cai et al. (65) investigated the mechanism of action of fermented seaweed extracts (FSE) on DKD. In STZ-induced DKD rats, the expressions of urine proteins, BUN, and SCr were markedly increased, while the administration of FSE could reverse their expressions. Furthermore, renal hypertrophy, accumulation of ECM, lobular changes in glomeruli, and thickening of the basement membrane occurred in DKD rats. The intervention of FSE could improve the above-mentioned pathological damage to varying degrees. In mechanism studies, FSE reduced mRNA and the protein expression of p-PI3K, p-AKT, and p-mTOR, respectively. The above-mentioned results suggest that FSE has the effect of alleviating renal injury in DKD and that the relevant mechanism may be associated with the modulation of the PI3K/AKT/mTOR signaling pathway.

In addition, *Dendrobium* mixture (66), Danggui Buxue decoction (67), and salvianolic acid B and tanshinone IIA (68) have markedly ameliorated DKD through mechanisms anchored in suppressing the PI3K/AKT signaling pathway.

### 2.6 Other signaling pathways

It was mentioned above that hirudin could improve DKD by intervening in the p38 MAPK/NF-κB signaling pathway. Pang et al. (69) also reported the intervention effect of hirudin on DKD, which was similar to the research results of Han et al. (54). Hirudin reduced blood glucose, SCr, UTP, and urea nitrogen levels in diabetic rats. In HE, PAS, and Masson’s trichrome analysis, hirudin improves mesangial stromal hyperplasia, mesangial cell proliferation, and glomerular sclerosis in the kidneys of rats and vacuolar degeneration of the tubule epithelial cells in diabetic rats. In further mechanism studies, hirudin reduced the expression of ECM-associated proteins fibronectin and type IV collagen and inhibited the expression levels of HIF-1a and VEGF in the renal tissues of diabetic rats. These findings indicate that hirudin could downregulate the expression of ECM markers in DKD renal tubular epithelial cells by inhibiting the HIF-1α/VEGF signaling pathway, thereby contributing to DKD amelioration.

TangShenWeiNing formula (TSWN) is a hospital preparation that has been used in clinical practice for over 20 years and has a positive impact on reducing proteinuria and protecting podocytes in patients with DKD (70). Chang et al. (70) found that TSWN reduced the levels of urinary albumin excretion (UAE) and urine albumin-to-creatinine ratio (UACR) in the kidneys of db/db mice. It may improve renal fibrosis and mesangial basement membrane thickening as well as increase mesangial glomerular basement membrane and KW nodule formation in db/db mice. Mechanistically, TSWN increased SIRT1, podocin, collagen I, and cleaved caspase-3 and inhibited the level of HIF-1α in renal tissues of db/db mice. The above-mentioned findings suggest that TSWN exerts a DKD-improving effect, and its mechanism may be associated with the SIRT1/HIF-1α signaling pathway.

Fructus arctii (FA) is the dried ripe fruit of *Arctium lappa* Willd which belongs to the Asteraceae family. It has anti-inflammatory, antioxidant, and anti-diabetic effects. Its anti-diabetic effect has been widely studied for many years, and it is one of the key members of various TCM prescriptions for treating diabetes (71–73). Zhang et al. (73) found that FA could reduce KW, blood glucose, proteinuria, SCr, and BUN levels in db/db mice. Furthermore, the kidneys of db/db mice showed glomerular structural abnormalities, mesangial matrix expansion, and collagen deposition. The mesangial matrix index and collagen area percentage and these renal pathological changes may show improvement following FA administration. Mechanistically, FA decreased the expression of fibronectin, collagen I, and α-SMA and increased the expression of Apoh and PPAR-γ. The above-mentioned results indicate that FA could inhibit the proliferation of mesangial cells and renal tubular cells and alleviate renal fibrosis through the Apoh/PPAR-γ signaling pathway, thereby playing a role in improving DKD.

In addition, catalpol (74) regulates the RAGE/RhoA/ROCK signaling pathway, Tang-Shen-Ning decoction (75) regulates the Wnt/β-catenin signaling pathway, Hederagenin (76) regulates the Smad3/NOX4/SLC7A11 signaling pathway, icariin (77) regulates the AR/RKIP/MEK/ERK signaling pathway, JinChan YiShen TongLuo formula (78) regulates the HIF-1α-PINK1-Parkin signaling pathway, naringenin (79) regulates the SIRT1/FOXO3a signaling pathway, Baoshentongluo formula (80) regulates the PINK1/Parkin signaling pathway, and Shen-Qi-Jiang-Tang granule (81) regulates the TNF signaling pathway to improve DKD.

## 3 Discussion

Before Western medicine entered China, TCM was the main means of disease prevention and treatment throughout its history and played an important role in protecting the physical health of the Chinese people. The holistic treatment concept of TCM is a theoretical system completely different from Western medicine. With TCM’s escalating appeal around the globe and its continued integration with Western medicine, scholars have become increasingly interested in the mechanisms of TCM in preventing and treating diseases, with a surge of investigations into TCM’s renoprotective impact on DKD in recent years (**Table 1**). It is noteworthy that NF-κB/NLRP3, TGF-β/Smad, Nrf2, MAPK, and PI3K/AKT signaling pathways stood out as recurrent focal points in the aforementioned TCM–DKD research. TCM counters DKD by targeting and resolving the critical pathological drivers of oxidative stress, autophagy, podocyte injury, apoptosis, and inflammation, which is consistent with the current mainstream findings on anti-DKD—for example, oxidative stress is recognized as central to the occurrence and development of DKD. HG levels can lead to an increase in ROS production via the polyol, PKC, and hexosamine pathways, thereby enhancing the oxidative stress response. In this way, NF-κB/NLRP3, TGF-β/Smad, Nrf2, PI3K/AKT, and MAPK signaling pathways are activated, which subsequently induce inflammation and form a malignant oxidative stress–inflammatory cycle. This leads to podocyte injury, extracellular matrix accumulation, epithelial–mesenchymal transition-related proteinuria, and other damages (82, 83). In this paper, AS-IV regulates the NF-κB/NLRP3 signaling pathway, baicalin regulates the MAPK signaling pathway, hirudin regulates the p38 MAPK/NF-κB signaling pathway, and SJF and HKC regulate the TLR4/NF-κB/NLRP3 signaling pathway to reduce the inflammation in DKD. The results indicate that the NF-κB signaling pathway plays a dominant role in improving the inflammation of DKD. Further in-depth studies of this signaling pathway, focusing on its cascading effect on DKD with other signaling pathways, will remain a central priority for forthcoming studies. In terms of anti-oxidative stress, the signaling pathways involved in the intervention of TCMs in this paper do not exhibit significant consistency—for instance, AS-IV regulates the NF-κB/NLRP3 signaling pathway, baicalin regulates the Nrf2 signaling pathway, LWDHP regulate the TGF-β/Smad, NF-κB, and MAPK signaling pathways, and Huangqi decoction regulates the TGF-β/MAPK/PPAR-γ signaling pathway to reduce the oxidative stress in DKD. Currently, research on the role of TCM in regulating autophagy and apoptosis in DKD has been a hot topic. However, the study focused on its pro-autophagy and anti-apoptotic effects and did not cover signaling pathways. As a result, the number of included signaling pathways is not large. Moreover, many of the TCMs in this paper play a role in improving DKD through multiple signaling pathways rather than a single one—for example, baicalin could inhibit inflammation in DKD by regulating the MAPK signaling pathway and oxidative stress in DKD by regulating the Nrf2 signaling pathway. AS-IV improves oxidative stress, inflammation, and podocyte injury of DKD by inhibiting the NF-κB/NLRP3 and MEK1/2-ERK1/2-RSK2 signaling pathways. LWDHP improves oxidative stress and fibrosis in DKD via intervening in TGF-β/Smad, NF-κB, and MAPK signaling pathways. FXG improves DKD by intervening in TGF-β1/Smad and VEGF/VEGFR2 signaling pathways. The multi-component feature of TCM also inherently endows it with the ability to exert effects at multiple targets and on multiple pathways. Due to the complexity of the pathogenesis and the long-term nature of DKD, TCMs with multiple targets, multiple pathways, and few adverse reactions are selected as drug candidates for the treatment of DKD with higher adaptability and broader prospects. Although TCM has shown many beneficial effects in the treatment of DKD, it still has some limitations that limit its current development. First, the principal constituents of TCM are constrained by the growing environment, leading to variable therapeutic outcomes and differences in toxic components, which also warrant attention. Second, hospital preparations account for a considerable proportion of TCM treatments for DKD, but they lack an explicit manufacturing process, which may lead to differences in therapeutic effects. Third, there is a lack of negative or positive control groups in some TCM experiments, and even studies only cover a single concentration of TCM. Finally, the gut microbiota constitutes a complex ecosystem composed of vast microbial communities, which establishes dynamic interactions with the host through modulating immune responses, nutrient absorption, metabolic transformations, and toxin degradation, thereby playing a pivotal role in preserving host health. Nevertheless, once the homeostasis of gut flora is disrupted, pathogenic bacteria proliferate extensively, while beneficial microorganisms diminish markedly. This process is accompanied by the accumulation of uremic toxins that subsequently accelerate the pathological progression of DKD. Alleviating DKD through microbiota modulation currently represents a highly active research frontier, with mounting evidence underscoring the critical contribution of intestinal dysbiosis to the pathogenesis and advancement of chronic kidney diseases, DKD included (84–87). It is notable that studies on how TCM regulates the gut microbiota to improve DKD have gradually emerged, such as geniposidic acid (88), acteoside-containing caffeic acid (89), and Yiqi Wenyang formula (90). However, very few of these studies have involved the study of the associated signaling pathways. Similarly, insulin sensitivity is one of the key metabolic signatures of DKD and is a major research focus for diabetes and its complications. However, there is a lack of research on the signaling pathways through which TCM interventions on insulin sensitivity improve DKD. Therefore, studying the signaling pathways related to gut microbiota and insulin sensitivity to improve DKD may be a very promising research direction in the future.

**Table 1:** *In vivo* and *in vitro* experimental evidence of TCM in the treatment of DKD.

Pathological progressions	Agents	Active compounds and plants	Experiment model		Molecular mechanisms	Signaling pathways	References
			In vivo	In vitro			
Oxidative stress, inflammation, podocyte injury	Astragaloside IV (AS-IV)	*Astragalus membranaceus*	SD rats, C57BL/6 mice	Podocytes	↓: Caspase-1, IL-1β, IL-18, GSDMD-N, NLRP3, ASC, p-p65, p65, UAlb, UACR, SCr, p-MEK1/2, p-ERK1/2, p-RSK2 ↑: klotho, nephrin, HDL-C	NF-κB/NLRP3, MEK1/2-ERK1/2-RSK2	(18, 51),
Inflammation	ShengQing Jiangzhuo formula (SJF)	*Astragalus membranaceus*, *Smilax glabra* Roxb., Rheum officinale, *Bombyx batryticatus*, *Periostracum cicadae*, *Rhizoma curcumae* Longae	db/db mice	–	↓: IL-1β, IL-18, TGF-β1, TLR4, p-p65, MyD88, NLRP3, Caspase-1, GSDMD-N, UTP, SCr	TLR4/NF-κB/NLRP3	(19)
Renal tubular epithelial-to-mesenchymal transition, inflammation	Huangkui capsule (HKC)	*Abelmoschus manihot* (L.) medic	SD rats	–	↓: Vimentin, collagen-1, a-SMC, NLRP3, Caspase-1, IL-1β, TLR4, p-IKK, p65	TLR4/NF-κB/NLRP3	(20)
–	Fuxin granules (FXG)	*Scrophularia ningpoensis* Hemsl, *Rheum palmatum* L., *Ramulus Euonymi*, *Astragalus propinquus* Schischkin, *Alisma plantago-aquatica* Linn, *Anemarrhena asphodeloides* Bunge, *Cuscuta chinensis* Lam	db/db mice	–	↓: BU, SCr, UCr, mALB, TGF-β1, p-Smad2/3, Smad2/3, VEGFA, VEGFR2 ↑: eNOS	TGF-β1/Smad, VEGF/VEGFR2	(26)
Oxidative stress, renal fibrosis	Liuwei Dihuang pill (LWDHP)	Prepared *Rehmannia* root, *Cornus officinalis*, Cortex Moutan, Rhizoma Dioscoreae, Poria Cocos, Alismatis Rhizoma	SD rats	–	↓: MDA, TGFβ-RI, TGFβ-RII, p-Smad2/3, α-SMA, NF-κB, p-p38, p-ERK ↑: SOD, NOS	TGF-β/Smad, NF-κB, MAPK	(28)
–	*Taxus chinensis* (TCN)	*Taxus chinensis*	SD rats	–	↓: BUN, SCr, UAER, TGF-β1, p-Smad2/3, α-SMA	TGF-β1/Smad	(31)
–	Shengqing Jiangzhuo capsule (SJC)	–	db/db mice	Human glomerular mesangial cell (HMC)	↓: KEAP1, SCr, urine microalbumin, AGEs, ROS, TC, TG, LDL-C ↑: Nrf2, γ-GCS, SOD1, HO-1, NQO1, GST, HDL-C	Keap1/Nrf2	(38)
–	Burdock fructooligosaccharide (BFO)	Burdock	C57BL/6J mice	–	↓: UTP, SCr, BUN, TC, TG, LDL-C, Bax ↑: HDL-C, SOD, CAT, Nrf2, HO-1, Bcl-2	Nrf2/HO-1	(41)
Oxidative stress, inflammation	Baicalin	*Scutellaria baicalensis* Georgi	db/db mice	–	↓: urine albumin level, ACR, AER, Bax, cleaved caspase-3, MDA, IL-1β, IL-6, MCP-1, TNF-α, p-Erk1/2, p-JNK, p-p38 ↑: Bcl-2, GSH-PX, SOD, CAT, Nrf2, HO-1, NQO-1	Nrf2, MAPK	(44)
Inflammation, apoptosis	Hirudin	Leeches	SD rats	Podocytes	↓: p-p38, p-p65, TNF-α, IL-1β, IL-6	p38 MAPK/NF-κB	(54)
Autophagy	Rutin	–	db/db mice	GEnCs	↓: α-SMA, Collagen1, HDAC1, p-PI3K, p-AKT, p-mTOR, p-S6K1 ↑: CD31, VE-cadherin, Syndecan-1 and Glypican-1, LC3BII/LC3BI ratio, Beclin1	PI3K/AKT/mTOR	(62)
–	Fermented seaweed extracts (FSE)	*Laminaria japonica*	Wistar rats	–	↓: urine proteins, BUN, SCr, p-PI3K, p-AKT, p-mTOR	PI3K/AKT/mTOR	(65)

↑, upgrade; ↓, downgrade.

In conclusion, as detailed in this review, a growing number of researchers have recognized the significance of TCM in the treatment of DKD. As research continues to deepen and expand, we suggest that, in the future, more efforts should be made to fill the gaps in the study of relevant TCM anti-DKD signaling pathways in emerging fields such as gut microbiota. This not only provides ideas and evidence for the development of new anti-DKD drugs but also provides more diverse options for the clinical use of TCM as complementary and alternative therapies.
